# Isolated palmar dislocation of distal radioulnar joint: a new mechanism of injury: a case report

**DOI:** 10.1186/s12891-019-2734-6

**Published:** 2019-08-09

**Authors:** Xianke Lin, Hui Shen, Hui Lu

**Affiliations:** 1 Department of Gastrointestinal Surgery, The First Affiliated Hospital, Zhejiang University, # 79Qingchun Road, Hangzhou, Zhejiang Province People’s Republic of China 310003; 2 Department of Orthopedics, The First Affiliated Hospital, College of Medicine, Zhejiang University, #79 Qingchun Road, Hangzhou, Zhejiang Province People’s Republic of China 310003

**Keywords:** Isolated palmar dislocation of distal radioulnar joint, Closed reduction, Misdiagnosis

## Abstract

**Background:**

Isolated palmar dislocation of distal radioulnar joint is a rare injury. It can easily lead to misdiagnosis. Previous literature reports were all rotation violence. We reported a patient with direct impact violence.

**Case presentation:**

We report a 31-year-old male laborer presented to our hospital with an acute trauma. Severe tenderness and limited mobility were seen in his right wrist. He received an x-ray film examination and diagnosed as the isolated palmar dislocation of distal radioulnar joint. The treatment was closed reduction and splint fixation. After half a year, the patient gained a functional recovery of his previously afflicted wrist.

**Conclusions:**

To the best of our knowledge, this is the first case of isolated palmar dislocation of distal radioulnar joint caused by a direct impact violence. Patients and physicians should be aware of the properties of this mechanism of injury so that early diagnosis and treatment can be achieved.

## Background

Isolated palmar dislocation of distal radioulnar joint (DRUJ) is not a common injury without concomitant fracture of the distal radius or ulna [1]. Emergency physician and orthopedic surgeon may easily miss this injury, so it will leave serious functional disability [2]. This kind of simultaneous opposition impact violence has existed only in the theory, and no actual case report have been reported. We first reported this injury mechanism, treatment and outcome.

## Case presentation

**Fig. 1:**
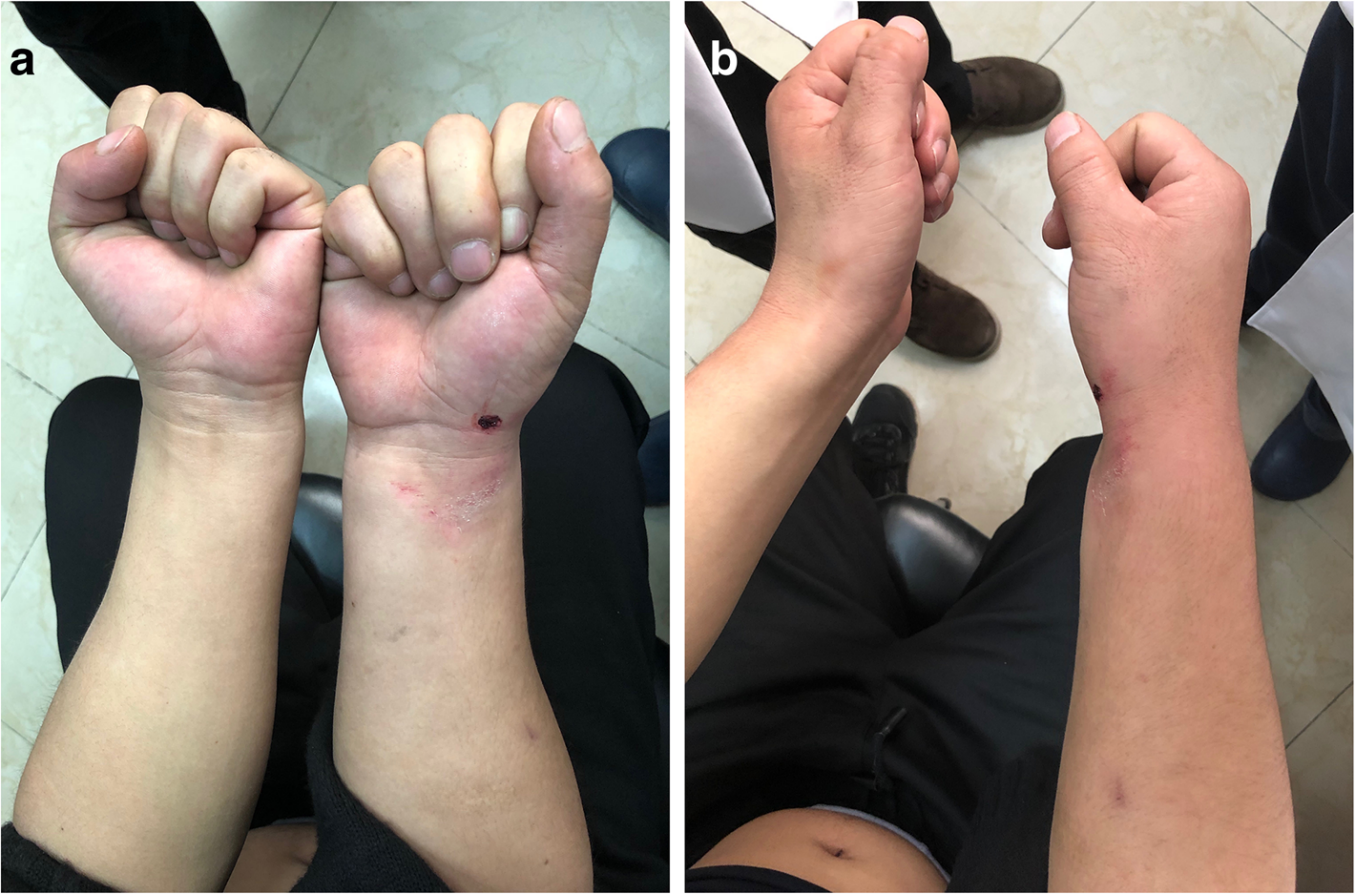
**a** Patient had local bruising on his radial palmar side of the of swelling wrist. **b** Abnormal bony prominences was seen on the ulnar palmar side

**Fig. 2:**
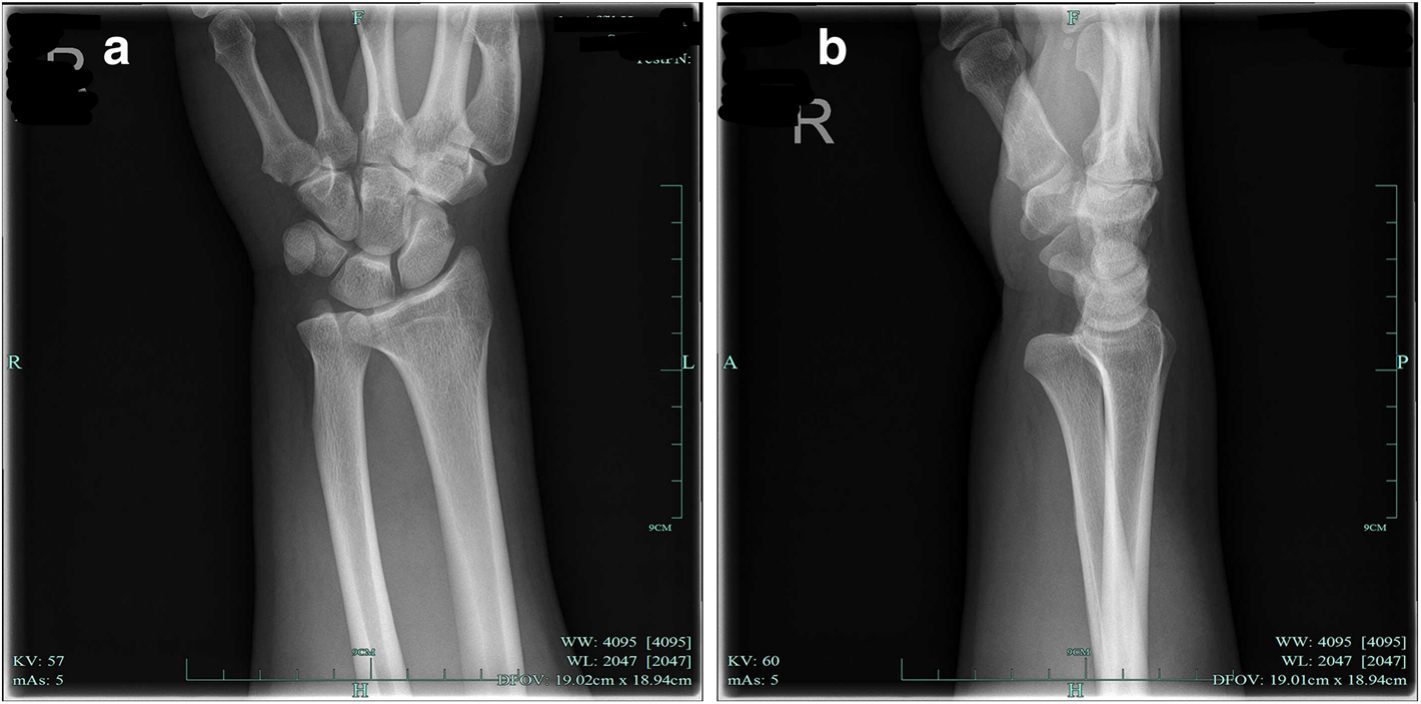
**a** Anteroposterior X-ray films showed overlap of the distal radius and ulna. **b** Lateral X-ray films showed palmar volar projection of the ulna relative to the radius

**Fig. 3:**
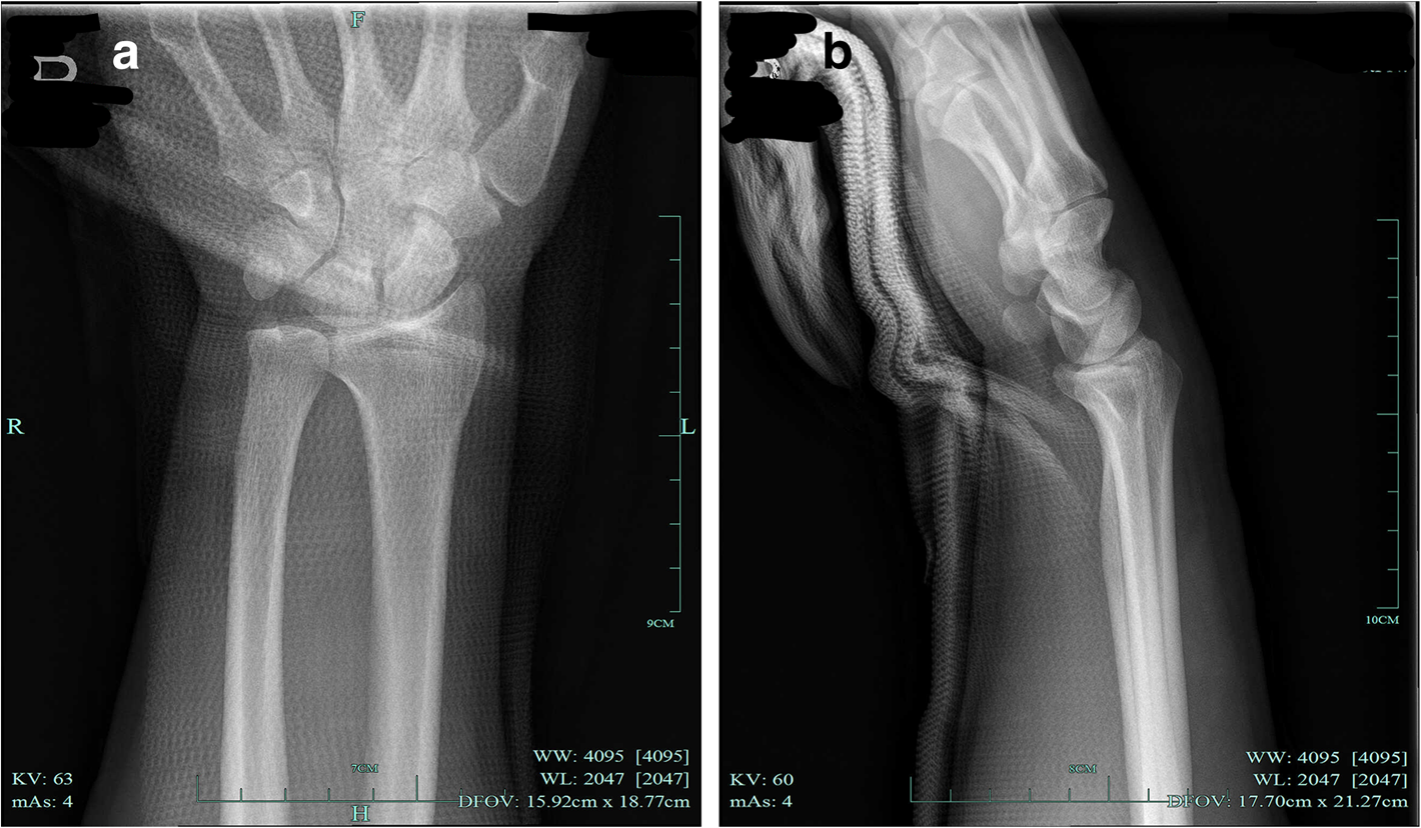
X-ray films reveals a complete reduction of dislocation (**a**) Anteroposterior X-ray films. **b** Lateral X-ray films

This is a case of a 31-year-old male laborer presented to our hospital with a direct impact trauma. He and his colleagues were installing outdoor units of air-condition. He lifts it with his right hand on the left side. Due to the unstable body, the machine was directly pressed against the radial palmar surface of his wrist, and the ulnar dorsal of his wrist hit the edge of the window sill. He felt immediate acute severe pain. The patient had no previous medical or surgical history related to the injury, and had no previous injuries to the wrist, forearm or hand. Physical examination revealed local bruising on the radial palmar side of the wrist, and abnormal bony prominences on the ulnar palmar side. The ulnar styloid was not palpable on the ulnar dorsal side of the wrist (Fig. 1). Movement of wrist was limited, movement of fingers was normal, there was no paresthesia in the fingers, and neurological function was normal. Plain X-ray films documented isolated palmar dislocation of DRUJ with soft tissue swelling. Anteroposterior X-ray films showed overlap of the distal radius and ulna. Lateral X-ray films showed palmar volar projection of the ulna relative to the radius (Fig. 2). In such a severe dislocation, we recommend magnetic resonance imaging to assess the injury of ligament, joint capsule and triangular fibrocartilage complex (TFCC). The patient refused to undergo examination and open surgery because of economic reasons. We underwent closed reduction under brachial plexus block. We used the thumb to directly press the palmar ulnar side of wrist, without rotating the wrist, and successfully reduction after hearing a sound of click. The patient’s right wrist did not dislocate again when it rotated 45 degrees of pronation and supination. Post-reduction films showed a complete reduction of dislocation (Fig. 3). A above elbow splint was used for one and half a month. The patient refused to take pain medicine and relief swelling medicine. The patient then performed normal daily work after 3 months. In a telephone follow-up 6 months later, he expressed satisfaction with his wrist function. He returned to the previous heavy physical activity. These study protocols were approved by the Medical Ethics Committee of the First Affiliated Hospital, College of Medicine, Zhejiang University.

## Discussion and conclusions

**Fig. 4:**
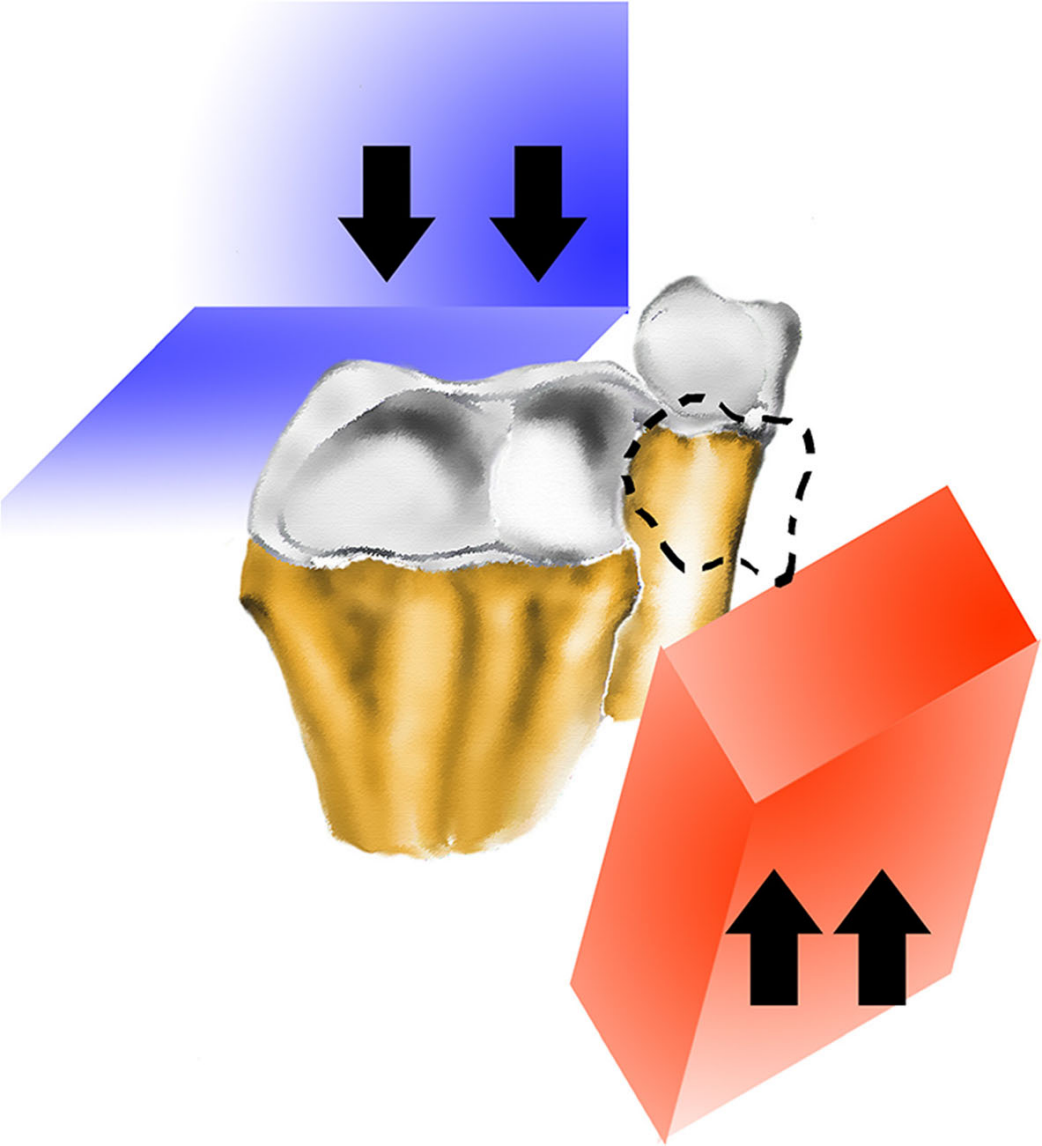
The diagram of injury mechanism

In the literature, the injury mechanism of Isolated palmar dislocation of DRUJ is mainly forced hyper supination of the forearm [3–6], fall from height [7–10] and intense impact sports activities [7, 11, 12], such as football (Table 1). As mentioned above, wrist rotation and great violence are the main factors, but our case is direct impact violence, impact on opposition (Fig. 4). The weight of machine and the impact of window sill edge were both direct violence. This situation is described for the first time. We think that although it was very rare, clinical radiologists, orthopedists, and hand surgeons must understand this mechanism to prevent missed diagnosis. It is easy to miss diagnosis this trauma on the initial X-rays, especially if the lateral view was not well positioned. So, the standard lateral view or both wrists as a contrast of X-Ray film were significant for diagnosis. Ct scan is more intuitive, and MRI can evaluate the conditions of ligament, TFCC and the interosseous membrane, which maintain the stability of the DRUJ [13].

**Table 1:** Literature of Isolated Palmar Dislocation of Distal Radioulnar Joint

	Mechanism of Injury	injury time (fresh< 3 weeks,old> 3 weeks)	combined injury	treatment	outcome
Kameyama M,2000 [4]	twisting in rotating machinery, forcibly supinated and flexed volarly	fresh	extensor tendon rupture, posterior interosseous nerve	Closed reduction+ Secondary tendon repair	good
Bouri F,2016 [5]	using the electrical drill, the drill got stuck and his left forearm forcefully rotated in supination	fresh	N/A	Closed reduction	good
Schiller MG,1991 [6]	pulling a heavy object, with the forearm supinated, when the volar aspect of the distal part of the radius was struck by a pulley	old	N/A	closed reduction+ Steinmannpin Fixed	good
Rijal L, 2012 [7]	fall on outstretched hand	fresh	N/A	Closed reduction+ K-wire fixed	good
Kohyama S, 2014 [8]	fallen with wrist supinated	old	avulsion of the TFCC, rupture of the deep dorsal and volar radioulnar ligaments	open reduction + anchor suture	good
Kashyap S, 1991 [9]	fall on the outstretched hand	old	extensor carpi ulnaris	Open reduction+ K-wire Fixed+ecu reconstruction	good
Mittal R, 2004 [10]	fall on his outstretched hand	fresh	N/A	Closed reduction	good
Mcmurray D, 2008 [11]	playing rugby	fresh	none	Closed reduction+ K-wire fixed	good
Kumar A, 1999 [12]	playing rugby. Come down heavily on his left hand and twisted his forearm	fresh	N/A	Closed reduction	good
Francobandiera C, 1990 [14]	injured wrist while training	old	ruptured triangular fibro-cartilage complex	TFCC partially excised	good
Singletary EM, 1994 [15]	tripped	fresh	N/A	Closed reduction	N/A

For fresh injury, patients combined with tendons or nerves injury were easy for an emergency doctor or orthopedic surgeon to notice [4]. For old injury, patients often have limited functional limitations and joint stiffness, so ct scan and MRI were available to help diagnosis [8, 9, 14] (Table 1). Due to economic reasons and wrist swelling at emergency injury, we cannot evaluate these structures such as ligaments. The volar radioulnar ligament may be ruptured, according to the weakness of the palmar soft tissue and pressure of the ulna during reduction.

The treatment generally depends on the injury mechanism, especially the closed reduction. Closed reduction, Kirschner wire fixation and cast immobilization were used when fresh injured or emergency-department visits [5, 7, 10–12, 15] (Table 1). Open reduction and reconstruction of ligament or TFCC were used when old injury or misdiagnosis [4, 8, 9, 14]. Although the overall number of cases is small, whether it is fresh injury or old injury, the final reported treatment results are satisfactory. For patients who have recurrent dislocation after reduction, it is very important to reconstruct the stability of DRUJ. Modified Sauve-Kapandji procedure is an option [16]. In our case, we press the prominent palmar side of ulnar head directly to successfully reduction, instead of pronating the hand like most of the time. Due to prompt diagnosis and treatment, although our patient received no ligament repair, he also achieved good wrist motion and function after splint fixation.

The direct, opposition impact violence is a rare injury mechanism that causes isolated palmar dislocation of DRJU. Clinicians must have enough knowledge and understanding of it. The treatment aim to the injury mechanism is more effective.

## Data Availability

The dataset supporting the conclusions of this article is included within the article.

## Abbreviations

DRUJ: Distal radioulnar joint
MRI: Magnetic resonance imaging
TFCC: Triangular fibrocartilage complex
